# Prediction of chronic damage in systemic lupus erythematosus by using machine-learning models

**DOI:** 10.1371/journal.pone.0174200

**Published:** 2017-03-22

**Authors:** Fulvia Ceccarelli, Marco Sciandrone, Carlo Perricone, Giulio Galvan, Francesco Morelli, Luis Nunes Vicente, Ilaria Leccese, Laura Massaro, Enrica Cipriano, Francesca Romana Spinelli, Cristiano Alessandri, Guido Valesini, Fabrizio Conti

**Affiliations:** 1 Lupus Clinic, Rheumatology, Dipartimento di Medicina Interna e Specialità Mediche, Sapienza Università di Roma, Rome, Italy; 2 Dipartimento di Ingegneria dell'Informazione, Università di Firenze, Florence, Italy; 3 Departemento de Matematica, Universidade de Coimbra, Coimbra, Portugal; Keio University, JAPAN

## Abstract

**Objective:**

The increased survival in Systemic Lupus Erythematosus (SLE) patients implies the development of chronic damage, occurring in up to 50% of cases. Its prevention is a major goal in the SLE management. We aimed at predicting chronic damage in a large monocentric SLE cohort by using neural networks.

**Methods:**

We enrolled 413 SLE patients (M/F 30/383; mean age ± SD 46.3±11.9 years; mean disease duration ± SD 174.6 ± 112.1 months). Chronic damage was assessed by the SLICC/ACR Damage Index (SDI). We applied Recurrent Neural Networks (RNNs) as a machine-learning model to predict the risk of chronic damage. The clinical data sequences registered for each patient during the follow-up were used for building and testing the RNNs.

**Results:**

At the first visit in the Lupus Clinic, 35.8% of patients had an SDI≥1. For the RNN model, two groups of patients were analyzed: patients with SDI = 0 at the baseline, developing damage during the follow-up (N = 38), and patients without damage (SDI = 0). We created a mathematical model with an AUC value of 0.77, able to predict damage development. A threshold value of 0.35 (sensitivity 0.74, specificity 0.76) seemed able to identify patients at risk to develop damage.

**Conclusion:**

We applied RNNs to identify a prediction model for SLE chronic damage. The use of the longitudinal data from the Sapienza Lupus Cohort, including laboratory and clinical items, resulted able to construct a mathematical model, potentially identifying patients at risk to develop damage.

## Introduction

Systemic lupus erythematosus (SLE) is a chronic autoimmune disease characterized by a multifactorial pathogenesis in which genetic and environmental factors interplay, determining disease development [1]. The production of a wide range of autoantibodies is a disease hallmark, leading to different clinical phenotypes [2]. The survival of SLE patients dramatically changed in the last 60 years, moving from the 50%, described in the 1950s, to the current over 90% [3]. The increased survival determined the possible accrual of chronic damage, related to adverse events of treatment, disease activity and comorbidities [4–6]. In order to quantify the damage in SLE patients and to measure over time modifications, the Systemic Lupus Collaborating Clinics (SLICC) and the American College of Rheumatology (ACR) proposed and validated a specific index, the SLICC/ACR Damage Index (SDI) [7]. Studies using such index in SLE cohorts demonstrated that damage accrual is associated with several demographic and clinical features including age and disease duration. Moreover, the presence of specific lupus-associated autoantibodies, such as anti-phospholipid antibodies (aPL), seems to be associated with damage development as well as disease activity, in particular, the occurrence of flares [8]. Nonetheless, some treatments such as glucocorticoids and immunosuppressive agents, despite their role in disease management, could intervene in determining chronic damage [4, 9].

However, despite an earlier diagnosis and the improvement of therapeutic strategies, the development of chronic damage represents a frequent event in SLE patients. A recent analysis of our Sapienza Lupus Cohort showed the presence of an SDI ≥1 in 35.8% of patients after a mean disease duration of about 14 years [10].

The majority of SLE patients begins to accrue damage during the early stages of the disease and it increases overtime. This phenomenon has been associated with different factors, such as age and activity at the onset, sex, ethnicity, disease duration and early appearance of damage [4]. Data from the Hopkins Lupus Cohort on 2,054 prospectively evaluated SLE patients, demonstrated that SDI score increased at a rate of 0.13 per year. Moreover, in this cohort, older age at diagnosis, ethnicity, and low income were the most important demographic predictors of damage progression [9]. The recently published study conducted by Legge et al. showed an increase in the SDI score ≥ 1 in more than 40% of patients after a mean follow-up of seven years [11]. According to these evidences, the identification of new tools able to predict the accrual and the progression of SLE damage is a strategic goal in order to identify patients at higher risk.

In the last years, it has been suggested that Artificial Neural Networks (ANNs) could be a useful prediction tool in medical *scenarios*. Such mathematical models express complex relationships between input and output data mimicking the human neural architecture of the brain and have been used in different ways in order to learn the relationship between a set of inputs and their outputs [12]. In the medical application, patients’ data could be considered as inputs and the specific outcomes as outputs. The *supervised training* procedure consists in tuning the parameters (*weights*) of the ANN model to produce the desired outputs using a set of training examples. Each example consists of some data (set of *features*), used as input to the network, and a *label*, which is what the network must learn to reproduce. The weights are modified iteratively until the output of the network for each training example is sufficiently close to its label. A trained ANN should be able to provide the correct labels in correspondence to new input data never used during the training process (*generalization capability*) [12, 13].

Few previous studies suggested that ANN could predict specific outcomes in SLE cohorts [14–17].

Focusing on renal involvement, neural network approach demonstrated an accuracy significantly higher compared with other methods in the LN prediction [14]. Moreover, ANNs were able to predict histological class, by identifying correlations between urinary protein spots and different parameters [15]. Machine-learning models was also applied to predict one-year outcomes of LN patients moving from baseline biomarker assessments [16]. Finally, these mathematical models could be applied in order to predict 3-year kidney graft survival in recipients affected by SLE [17].

Indeed, in the International Conference on Advanced Computing and Communication Systems, held in Coimbatore in 2015, it was underlined the possible application of sophisticated data analysis tools, such as machine learning methods, in SLE patients, in the light of their potential application to diagnostic and prediction purposes [18].

Moving from these premises, we aimed at evaluating whether the usage of ANN is able to predict the onset of damage in patients with SLE. Therefore, we employed these mathematical models as a model-based technique to analyze clinical and laboratory data deriving from the Sapienza Lupus Cohort.

## Materials and methods

We conducted a longitudinal study on adult SLE patients attending at the Sapienza Lupus Cohort. All patients satisfied the revised 1997 ACR criteria for SLE classification [19]. The local ethical committee of “Policlinico Umberto I/Sapienza Università di Roma” approved the study. Patients provided written informed consent at the time of the first visit at the Sapienza Lupus Clinic.

At each visit, the patients underwent a complete physical examination. Clinical and laboratory data were collected in a standardized, computerized, and electronically filled form, including demographics, past medical history with the date of diagnosis, co-morbidities, previous and concomitant treatments. All the patients were evaluated at least twice per year, even though most of the patients were observed quarterly. Selected patients could be followed more often, according to their clinical condition.

With regard to the laboratory assessment, antinuclear antibodies (ANA) were determined by indirect immunofluorescence assay (IIFA) on HEp-2, anti-dsDNA by IIFA on *Crithidia luciliae*, ENA (anti-Ro/SSA, anti-La/SSB, anti-Sm, anti-RNP), anti-cardiolipin (anti-CL) of IgG or IgM isotype and anti-Beta2glicoprotein I (anti-Beta2GPI) of IgG or IgM isotype by ELISA. Lupus anticoagulant (LA) was assessed according to the guidelines of International Society on Thrombosis and Hemostasis (ref). For all the subjects, complement C3 and C4 concentrations were determined by nephelometry (mg/dl).

Disease activity was evaluated at each visit by using the SLE Disease Activity Index 2000 (SLEDAI-2K) [20] and for the purpose of the ANN considered as a binary instance as absent (SLEDAI-2K = 0) versus any level of activity (SLEDAI-2k≥1).

### Chronic damage

Damage was measured by SDI in all the available examinations. The SDI score was calculated based on organ damage that occurred after SLE diagnosis. According to the SDI, damage was assessed in 12 organ systems: ocular (range 0–2), neuropsychiatric (0–6), renal (0–3), pulmonary (0–5), cardiovascular (0–6), peripheral vascular (0–5), gastrointestinal (0–6), musculoskeletal (0–7), skin (0–3), gonadal (0–1), endocrine (0–1) and malignancy (0–2), with a possible maximum total score of 47. The damage, defined as irreversible impairment, had to be persistent for at least six months [7].

### Statistical analysis

Categorical variables are summarized as frequencies and percentages, while continuous variables are presented as means and standard deviation (SD) or median (range), if normally or non normally distributed, respectively. Mann-Whitney test was performed when appropriate. Univariate comparisons between nominal variables were calculated using chi-square test or Fisher’s exact-test where appropriate. P values less than 0.05 were considered significant.

### Artificial neural networks

**Fig 1:**
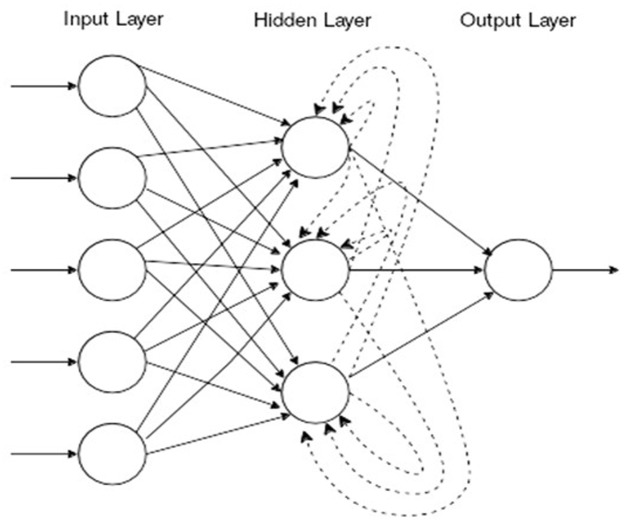
Illustration of a Recurrent Neural Network (dashed lines are backward).

We designed an ANN as a machine-learning model to predict the risk to develop chronic damage in SLE patients. In particular, for the aim of the present study, we employ Recurrent Neural Networks (RNNs) as model suited to deal with sequential information. This represents a neural network model suitable for sequential inputs. Specifically, the input data consisting of a sequence of sets of features are processed by the network one-step at a time through a series of *layers*: the input layer which receives the external inputs, a hidden layer, and an output layer which contains the outputs of the network. Each layer is composed of several units called *neurons* whose value depends on the connections with the other neurons. All layers are connected in a forward manner except for the hidden layer, which presents also a special backward connection like depicted in Fig 1. This special backward connection introduces a recurrence in the model, which is employed by the network to “remember” the information of the previous time steps (Fig 1).

At each time step, the network produces an output, but often, only the output of the last time step is considered. The labels associated with the training sequences can be sequences themselves, if we are interested also in the intermediate outputs of the network, or a single value only for the last step. It is important to remark that RNNs deal with sequences of arbitrary length and that the sequences that constitute the training examples do not need to have the same length. This makes RNNs an extremely versatile model. Therefore, for the aim of the present study, the sequences of clinical data registered for each SLE patient in the standardized, computerized and electronically-filled form during the follow-up have been used for building and testing the RNNs.

## Results

Four hundred and thirteen patients were enrolled consecutively in the present study (M/F 30/383; mean age ±SD 46.3±11.9 years; mean disease duration ±SD 174.6±112.1 months; Ethnicity: Caucasian 97.3%, Asian 1.7%, Latino-American 1.0%). Referring to the disease history, joint and skin involvement and hematological manifestations were the most frequent, occurring in 67.1%, 66.3% and 63.9% respectively. Patients were followed in the present outpatient clinic for a mean period ±SD of 63.9±30.7 months.

With regard to other associated systemic autoimmune diseases, 61 patients (14.8%) had anti-phospholipid syndrome (APS) and 28 (6.8%) Sjögren’s Syndrome (SS). Concerning other comorbidities not included in the SDI, treatment-required fibromyalgia and arterial hypertension were the most frequent (8.2% and 7.7%, respectively).

At the time of the first visit in our Lupus Clinic, 148 patients out of 413 (35.8%) had an SDI≥1, with a mean±SD value of 1.7±1.1 (median 1.0, range 1–8, 95% CI 3.0–4.0). Eighty-eight out of 148 patients (59.4%) showed an SDI = 1, 38/148 patients (25.7%) SDI = 2, 10/148 patients (6.7%) SDI = 3, 10 patients (6.7%) SDI = 4, one patient (0.7%) SDI = 7 and another one (0.7%) SDI = 8. During the observation period (mean ±SD 63.9±30.6 months, range 12–218), 66/413 SLE patients (15.9%) showed a progression of SDI score. When considering the distribution of damage according to the involved organ/system, neuropsychiatric and musculosketal involvement represent the most frequent in the present cohort, occurring both in 46 SLE patients (11.1%).

### Artificial neural networks

For the machine-learning model, we selected two groups: patients with SDI = 0 at the baseline, developing chronic damage during the follow-up (case, N = 38); patients without chronic damage (SDI = 0) at baseline who did not develop chronic damage. In particular, we used all the visits before a positive SDI was registered for patients in the first group. Concerning the second group, we considered patients with at least 5 visits and a successive follow-up of 2 years. We adopted this stringent selection in order to train and test the model with robust data with respect to (putative) negative patients. Without the adoption of the above criterion, the risk was of including in the negative instances patients controlled for a period too short to assign the label of negative patient.

Demographic, clinical and laboratory data of SLE cases and controls were reported in Table 1.

**Table 1:** Demographic features, clinical and laboratory manifestations and treatment of case (N = 38) and controls 8N = 94).

	CASES (N = 38)	CONTROLS (N = 94)	P-Values
**Demographic features**			
M/F	2/36	5/89	NS
Mean age ±SD (years)	43.4±10.0	35.6±10.9	0.0009
Mean disease duration ±SD (months)	126.0±97.2	87.6±80.4	0.03
**Ethnicity—N (%)**			
Caucasian	37 (97.4)	93 (98.9)	NS
Asian	1 (2.6)	1 (1.1)	NS
Latino-American	-		
**Clinical manifestations—N (%)**			
Joint involvement	26 (68.4)	62 (65.9)	NS
Skin involvement	21 (55.2)	65 (69.1)	NS
Serositis	1 (2.6)	14 (14.9)	0.002
Hematological manifestations	28 (73.8)	67 (71.3)	NS
Neuropsychiatric involvement	4 (10.5)	8 (8.5)	NS
Renal involvement	8 (21.0)	25 (26.6)	NS
**Laboratory manifestations—N (%)**			
Anti-DNA	21 (55.2)	58 (61.7)	NS
Anti-Sm	4 (10.5)	12 (12.8)	NS
Anti-SSA	10 (26.3)	29 (30.8)	NS
Anti-SSB	5 (13.1)	17 (18.1)	NS
Anti-RNP	8 (21.0)	15 (15.9)	NS
Anti-cardiolipin IgG/IgM	12 (31.6)	32 (34.0)	NS
Anti-β_2_Glycoprotein I IgG/IgM	10 (26.3)	14 (14.9)	NS
Lupus Anticoagulant	8 (21.0)	17 (18.1)	NS
Low C3 levels	14 (36.8)	32 (34.0)	NS
Low C4 levels	9 (23.7)	26 (27.6)	NS
**Treatments—N (%)**			
Corticosteroids	35 (92.1)	77 (81.9)	NS
Hydroxychloroquine	33 (86.8)	85 (90.4)	NS
Cyclosporine A	12 (31.6)	19 (20.2)	NS
Methotrexate	12 (31.6)	14 (14.9)	0.006
Cyclophosphamide	2 (5.3)	12 (12.8)	NS
Mycophenolate Mofetil	12 (31.6)	26 (27.6)	NS
Azathioprine	8 (21.0)	24 (25.5)	NS
Rituximab	2 (5.3)	3 (3.2)	NS
Belimumab	2 (5.3)	1 (1.1)	NS
ASA	21 (55.3)	37 (39.4)	0.03
Anticoagulant therapy	7 (18.4)	6 (6.4)	0.01
**Concomitant diseases and comorbidities—N (%)**			
Anti-phospholipid syndrome	4 (10.5)	10 (10.6)	NS
Sjögren’s Syndrome	6 (15.8)	6 (6.4)	0.03
Autoimmune thyroiditis	3 (7.9)	6 (6.4)	NS
Fibromyalgia	4 (10.5)	11 (11.7)	NS
Dyslipidemia	5 (13.1)	9 (9.6)	NS
Arterial hypertension	5 (13.1)	8 (8.5)	NS

**NS**: not significant.

As expect, we registered significantly higher mean age and disease duration values in the cases than in controls (43.4±10.0 *versus* 35.6±10.9 years, P = 0.0009; 126.0±97.2 versus 87.6±80.4 months, P = 0.03, respectively). Moreover, a concomitant Sjögren’s Syndrome was less frequent in SLE cases (P = 0.03).

We discarded all the binary features with less than four positive occurrences among all the patients. In Table 2, we reported the features used to build RNN model.

**Table 2:** Features used for the Recurrent Neural Network model.

Features
Sex
Age
Concomitant diseases (APS, Sjögren’s Syndrome, autoimmune thyroiditis, fibromyalgia)
Comorbidities (dyslipidemia and arterial hypertension)
Renal involvement
Skin involvement
Neurological involvement
Joint involvement
Hematological manifestations
Occurrence of arterial and/or venous thrombosis
Obstetrical complications
Autoantibodies positivity (anti-dsDNA, anti-SSA, anti-SSB, anti-Sm, anti-RNP, anti-Cl, anti-β2GPI, LA)
C3 and C4 serum level reduction
Disease activity (SLEDAI-2k)
Treatment during disease history (GC, HCQ, MTX, AZA, CyA, Cy, MMF, RTX, BLM)

GC: glucocorticoid, HCQ: hydroxychloroquine; MTX: methotrexate; AZA: azathioprine; CyA: Cyclosporine A; Cy: Cyclosphosphamide; MMF: mycophenolate mofetil; RTX: rituximab; BLM: belimumab.

The network we employed in this study was composed of 100 hidden units and organized as described by the following equations:

$$y_{t}^{j}=\frac{1}{1+exp\left(-W_{o}\;\cdot \;h_{t}^{j}\right)}$$

$$h_{t}^{j}=\;tanh\left(W_{r}\cdot \;h_{t-1}^{j}+W_{i}\cdot x_{t}^{j}\right)$$

$h_{0}^{j}=0$, where $x_{t}^{j}$ and $h_{t}^{j}$ are vectors, *W*_*r*_, *W*_*i*_, *W*_*o*_ are matrices and tanh is applied element-wise. If we denote by p be the number of features and by n the number of hidden units then h_t^jis a vector of lenth n andx_t^jof length p. As for the weight matrices, W ris n by n, W_jis p by n and W_ois n by 1.

**Fig 2:**
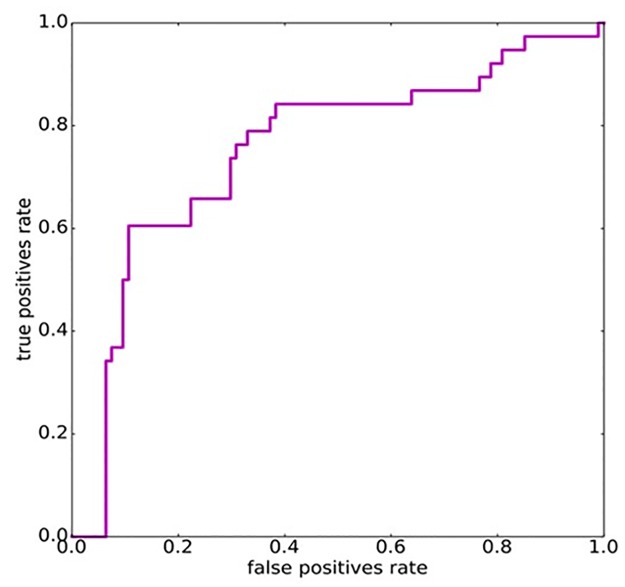
ROC curve for RNN model.

The input of the network is represented by the vector *x* and it is indexed by *j*, which identifies a patient, and *t* which loops over the time steps, namely, in our context, the visits of the patien. For instance $x_{1}^{1}$ is the vector of the features for the first visit of patient 1. The output of the network is *y* and, like *x*, is indexed in the temporal dimension by *t*, although in our case only the last value was considered. Note that, by definition, $y_{t}^{j}$ is a value in the interval [0, 1] and hence is interpreted as the probability of a patient to develop an organ/system damage in the next 2 years. The vector $h_{t}^{j}$, instead, expresses the values of the hidden units and it is used only as an intermediate step in the computation of the response $y_{t}^{j}$. It is interesting to notice how the preceding history of a patient is taken into account through the dependency of $h_{t}^{j}$ from $h_{t-1}^{j}$. The other quantities, *W*_*r*_, *W*_*i*_, *W*_*o*_ are the matrices which contain the weights of the connections which are tuned in order to produce the desired output. We used the Stochastic Gradient Descent (SGD) algorithm, one of the most commonly employed for ANNs training, to tune the weights of the network. We stopped the training procedure once the predictions for the training examples were sufficiently accurate, namely when the area under the ROC curve (AUC) was above 0.95. This technique, know as early stopping, is widely employed to avoid over-fitting. We evaluated the ability of the network to generalize with an eight-fold validation. We split the data, both positives, and negatives, in eight different parts and we iteratively trained an RNN using as a training set seven parts out of eight and computed the predictions on the leaved out part (test set). Once we had the predictions on all the eight parts we put them together and computed the true positive and false positives rates for increasing thresholds in the interval [0, 1] to obtain the ROC curve shown in Fig 2.

In order to assess the robustness of the model, we performed experiments by varying the architecture of the network, i.e., the number of hidden units. We did not observe substantial differences increasing the number of hidden units from 50 to 100. This is due to the effect of the early stopping criterion to avoid overfitting.

With this method, we observed that the area under curve (AUC) for the prediction of chronic damage was 0.77. In Table 3, we reported threshold values, which yield both sensitivity and specificity equal to 0.7 or higher.

**Table 3:** Threshold and the corresponding sensitivity and specificity values.

Threshold	Sensitivity	Specificity
0.486	0.819	0.711
0.383	0.755	0.711
0.365	0.745	0.737
**0.358**	**0.745**	**0.763**
0.290	0.702	0.763
0.271	0.702	0.789

According with the best sensitivity and specificity values, a threshold value of 0.35 could identify patients developing chronic damage. Finally, we performed further experiments by Feed-Forward Neural Networks (FFNNs) and by the common logistic regression approach. More specifically, we considered a "static" model, where the input of the network is a vector whose components are the features of the patient in the last L (> = 1) visits up to the second to last, available visit. In this way, we build a model with a single input vector that contains patient features across multiple time points. With L = 1, 2, 3, both FFNNs and the logistic regression approach obtained AUC slightly greater than 0.5. These results point out the advantages of using, for the specific predict task of the work, RNN that can deal with sequences of inputs.

## Discussion

To the best of our knowledge, this is the first study aimed at developing an RNN model to predict chronic damage in a large SLE population-based data.

We used the longitudinally recorded data from the Sapienza Lupus Cohort, including laboratory and clinical features, in order to apply the machine-learning model to predict SLE damage development. The selected items resulted able to construct a mathematical model characterized by a good performance, as demonstrated by the AUC value, higher than 0.7.

In the last years, several studies have suggested the possibility to apply mathematical systems in the medical sciences, in order to create models able to predict a specific outcome. Among these, ANN which mimicking biological neural networks could be trained in order to recognize underlying patterns of diseases. ANNs could simulate the neuron functions in order to process data and to learn from the experiences. After an appropriate training, neural networks could develop a higher accuracy in comparison with conventional classification analysis. More recently, the application of ANNs in medical decision support systems has been suggested, thanks their ability to detect complex nonlinear relationships between predictors and diseases [21–23]. Moving from real cases, the neural system can be trained in order to discover the relationships between different variables and to learn which features of the inputs are mostly related to the output [23].

Some studies evaluated the possible application of machine-learning models in SLE cohorts, focusing on kidney involvement. Rajimehr et al. published the first study on this topic in 2002: the neural networks efficiency for lupus nephritis (LN) prediction was compared with a logistic regression model and with clinicians’ diagnosis. Neural network approach showed an accuracy significantly higher compared with other methods, especially in predicting LN [14]. The study published by Oates and colleagues in 2005 aimed at applying ANNs to identify correlations between urinary protein spots, identified by gel electrophoresis, and different parameters in a cohort of 20 SLE patients with renal involvement undergoing renal biopsy. The output considered in this study was the binary value for each histological class according to the ISN/RPS classification, and an ordinal value for activity and chronicity indices. The input data were analyzed by the ANNs to determine their ability to predict the disease. For all histological classes, a sensitivity higher than 86% was identified, with a specificity of 92%. The ROC of the trained networks demonstrated an AUC value ranging from 0.85 to 0.95. The results of the study suggest the possibility to apply ANNs in order to identify a list of protein spots useful to develop a clinical assay able to predict ISN/RPS class and chronicity for LN patients, potentially replacing the renal biopsy [15].

More recently, the same research group aimed at applying machine-learning models to predict one-year outcomes of LN patients moving from baseline biomarker assessments. The urine samples from 140 biopsy-proven LN patients were collected prior to the induction treatment; numerous traditional and novel biomarkers were analyzed. The outcome variable considered in the study was complete remission after 12 months. For each biomarker, a ROC curve was generated by traditional statistical analysis and these were compared with AUC values obtained from machine learning models developed using random forest (RF) algorithms. A significantly greater AUC (0.79) was observed in the combined models in comparison with models created with traditional clinical markers alone. Moreover, the RF combined model had a significantly better AUC than the majority of the individual biomarkers, determining an improvement in terms of sensitivity [16]. In 2011, Tang and colleagues applied machine-learning models in order to predict 3-year kidney graft survival in recipients affected by SLE. Data from 4,754 SLE transplant recipients were obtained: among these 3,313 were followed for a 3-years period and included in the study. Thirty-eight variables were selected to perform three different classification methods: classification trees, artificial neural networks, and logistic regression. In particular, with regard to the neural networks, a feed-forward multilayer perceptron architecture was used, a model including an input layer, a single hidden layer—calculating the sum of weighted input predictors—and an output layer—producing the predicted probability of class membership. An AUC value of 0.71 was obtained by the application of ANN. Moreover, the performance of logistic regression and classification trees were not inferior to more complex artificial neural network. The authors of the study concluded that different prediction models could be used in clinical practice to identify patients at risk of the poorer outcome [17].

Other possible applications in SLE cohorts have been suggested. The study conducted by Ward and colleagues in 2006 suggested the use of RF to predict short-term mortality in a cohort of 3,839 hospitalized SLE patients. Among these, 109 patients died during hospitalization. The RF demonstrated high predictive accuracy for classification of death, identifying Charlson Index, respiratory failure, SLE Comorbidity Index, age, sepsis, nephritis, and thrombocytopenia as the most important predictors of mortality [24].

In the present study, for the first time, we applied the neural-network analysis to generate a prediction model for the chronic damage in patients affected by SLE.

Moving from a large monocentric SLE data set longitudinally evaluated a stringent selection of patients and of features inserted in the model has been performed, in order to safeguard the results power.

In particular, as cases we considered only patients without damage at the first visit developing it during the follow-up. Moreover, as controls, we selected only those patients without chronic damage at baseline who did not develop chronic damage in a follow-up period higher than 2 years with at least 5 visits. Even though such stringent selection reduced the number of available data (patients) used to train the neural network-based model, we obtained promising results.

Moreover, the computational study confirms that the recorded medical data contain information useful to predict damage development in SLE patients. A large number of features have been evaluated by a longitudinal approach in the Sapienza Lupus Cohort and these variables resulted able to predict damage development. With regard to clinical manifestations, in the RNN model all the clinical features considered in the 1997-revised ACR criteria have been included (namely renal and neurological involvement, articular, skin and hematological manifestations, serositis) [19]. Moreover, disease activity assessment was considered in the model, with the inclusion of SLEDAI-2K index and serological activity biomarkers (in particular anti-dsDNA antibodies and C3/C4 serum levels). Nonetheless, we inserted in the model some clinical manifestations—occurrence of arterial and/or venous thrombosis; obstetrical complications—potentially related to aPL positivity. This appears very interesting in the light of a large number of studies identifying an association between the damage accrual and the presence of these autoantibodies in SLE patients [8].

Moreover, among the comorbidities, the presence of an associated APS have been considered, in addition to Sjögren’s Syndrome, autoimmune thyroiditis, fibromyalgia, frequently identified in SLE patients.

Nonetheless, comorbidities, such as hypertension and dyslipidemia were also included in the model.

We chose to adopt the RNNs model because it seem to be suitable to deal with the prediction task object of the present work. Moreover, this specific model allows the evaluation of data deriving from sequential visits in the same patients.

In the present analysis, similarly to the others applying neural-network models, the AUC was chosen as the primary measure to evaluate a model’s discriminative power because it does not depend on the prediction threshold chosen for a model. We obtained an AUC value of 0.77, indicating a good performance of our model. We believe that significant improvements in the prediction performance of the RNNs could be obtained by using a larger number of training data. Moreover, we suggested the possibility to use a cut-off value, with good performance in terms of sensitivity and specificity, identifying patients at risk to develop chronic damage, moving from the baseline condition.

In conclusion, in the present study, we applied for the first time a machine-learning analysis in order to create a model able to predict chronic damage development in SLE patients. Our results suggest that moving from a core-set of clinical and laboratory features, it is possible to create a mathematical model able to predict chronic damage. This prediction tool could be used potentially in a clinical practice setting to stratify SLE patients according to the risk of developing chronic damage. The model we designed is a “black box” model for finding complex and implicit relationships between clinic features and SLE damage. The model was trained and tested using all the selected features. The identification of contributor features relevant for the model could be very interesting in order to better understand the pathogenic mechanisms involved in damage development. To this aim, standard feature ranking techniques of the literature, computing a score for each risk factor, could be applied. However, the available data for the present work are not sufficient to perform a reliable study along this direction and to draw sound conclusions. Larger cohorts are needed to test risk factor ranking issue to predict damage in SLE.
